# Characterization of Amino Acid Substitution W20S in MgrB Involved in Polymyxin Resistance in Klebsiella pneumoniae

**DOI:** 10.1128/spectrum.01766-21

**Published:** 2022-02-16

**Authors:** Mélanie Roch, Willames M. B. S. Martins, Roberto Sierra, Ana C. Gales, Diego O. Andrey

**Affiliations:** a Department of Microbiology and Molecular Medicine, Faculty of Medicine, University of Geneva, Geneva, Switzerland; b Service of Infectious Diseases, Department of Medicine, Geneva University Hospitals and Medical School, Geneva, Switzerland; c Department of Medical Microbiology, Division of Infection and Immunity, Cardiff University, Cardiff, United Kingdom; d Universidade Federal de São Paulo - UNIFESP, Division of Infectious Diseases, Department of Internal Medicine, Escola Paulista de Medicina - EPM, São Paulo, Brazil; e Division of Laboratory Medicine, Department of Diagnostics, Geneva University Hospitals and University of Geneva, Geneva, Switzerland; Emory University School of Medicine

**Keywords:** colistin, *Enterobacterales*, KPC, multidrug resistance

## Abstract

In the major human pathogen Klebsiella pneumoniae, MgrB inactivation by disruptive insertion sequence (IS) elements and mutations leading to early termination are known to play an important role in polymyxin resistance. In this study, we examined a collection of invasive *bla*_KPC-2_-producing K. pneumoniae isolates belonging to the high-risk clone sequence type 258 (ST258) displaying high rates of resistance to many antimicrobials, including polymyxins. We identified a deleterious substitution (W20S) in MgrB and confirmed by genetic complementation analysis that this variant was inactive, leading to increased polymyxin B and colistin MICs.

**IMPORTANCE** Carbapenem-resistant Gram-negative bacteria are designated critical pathogens by the World Health Organization. Polymyxins (i.e., polymyxin B and colistin) are last-resort antibiotics and particularly useful against these multidrug-resistant bacteria. In Klebsiella pneumoniae, the inactivation of MgrB, a negative regulator of PhoPQ, was shown to be the major pathway leading to colistin resistance. While gene disruption by insertion sequence (IS) elements and mutations leading to early termination (stop codons) are frequent, deleterious mutations are not observed frequently and have not been characterized. Here, we identified a deleterious substitution (W20S) in MgrB among a collection of bloodstream infection, *bla*_KPC-2_-producing K. pneumoniae sequence type 258 (ST258) isolates, displaying high rates of resistance to polymyxins and associated with a high mortality rate. The dissemination of such a MgrB-W20S mutation leading to polymyxin resistance within the ST258 high-risk clone background is problematic and thus warrants particular attention.

## OBSERVATION

Polymyxins are last-resort antibiotics, particularly against carbapenem-resistant Gram-negative bacteria. This family of polycationic antimicrobial peptides includes polymyxin B and polymyxin E (i.e., colistin). In Klebsiella pneumoniae, it is well established that the most frequent mechanism of polymyxin resistance is the inactivation of chromosomally encoded *mgrB.* The small (47 amino acids) membrane protein MgrB is a negative regulator of the two-component system PhoPQ that controls lipopolysaccharide (LPS) modifications. MgrB prevents PhoPQ hyperactivation by directly interacting in the membrane with the PhoQ sensor kinase, while unfunctional or the absence of MgrB leads to enhanced PhoPQ activity and downstream addition of 4-amino-4-deoxy-l-arabinose (l-Ara4N) on lipid A decreasing the LPS negative charge (1). MgrB inactivation has been shown to arise by (i) gene interruption by an insertion sequence (IS) element, (ii) nucleotide deletion/insertion leading to frameshift and premature stop codons, or (iii) nucleotide nonsense substitution creating a premature stop codon (2, 3). Here, we identified for the first time a single amino acid substitution (W20S) responsible for MgrB inactivation increasing polymyxin MICs in carbapenemase *bla*_KPC-2_-producing K. pneumoniae sequence type 258 (ST258) invasive isolates.

We recently described the clinical and epidemiological features associated with a cohort of 165 polyclonal *bla*_KPC-2_-producing K. pneumoniae bloodstream infections in a Brazilian tertiary hospital between 2014 and 2016 (4). Among the 42 *bla*_KPC-2_ isolates belonging to the sequence type 258 (ST258), we observed unexpectedly high rates of resistance to colistin (MIC_50_, 8 μg/mL; MIC_90_, 128μg/mL; 80% resistant). ST258 *bla*_KPC-2_ isolates carried multiple resistance genes, including *rmtB* aminoglycoside 16S-methylase, severely limiting therapeutic options (see Table S1 in the supplemental material) (5). The clinical burden of these 42 ST258 K. pneumoniae bloodstream infection cases is described in Table S1 (Hospital Sao Paulo/Universidade Federal de São Paulo Ethics Committee for Clinical Research protocol 1.814.158). Overall (all-cause) 30-day mortality was 59.5%. The low number of polymyxin susceptible isolates precluded a deeper analysis of the impact of polymyxin resistance on the outcome.

**FIG 1:**
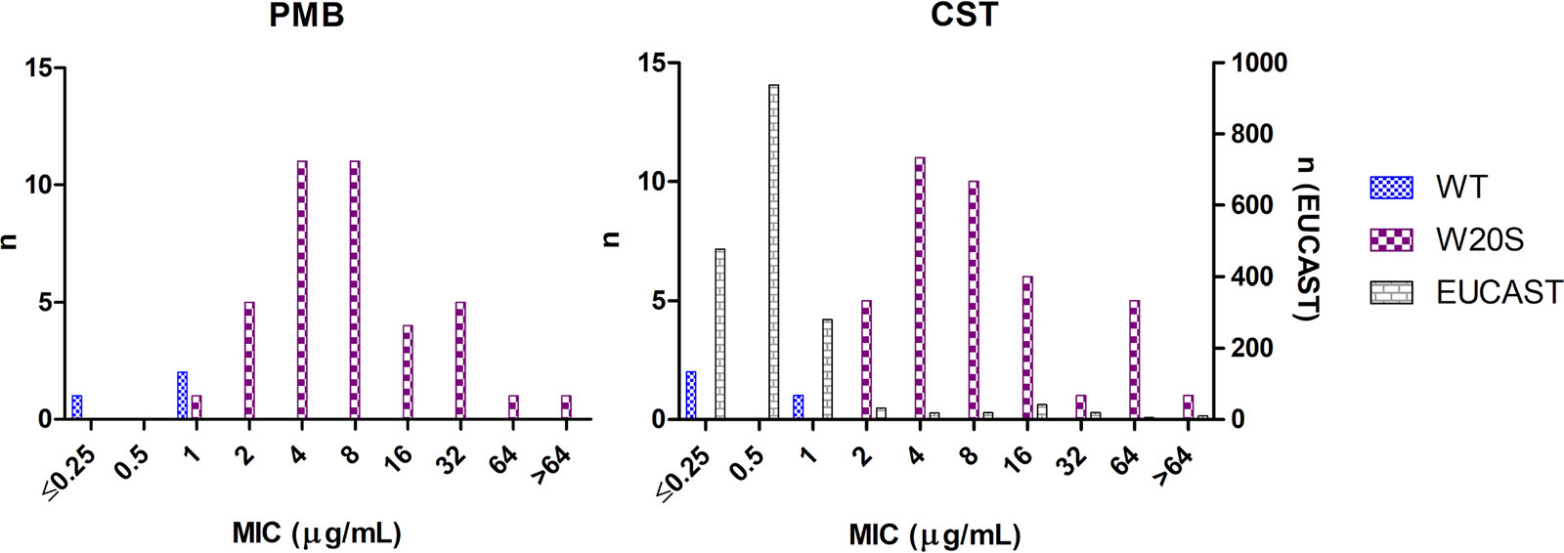
Polymyxin B and colistin MIC distribution of K. pneumoniae ST258 isolates exhibiting wild-type (WT) *mgrB* (blue) and a W20S *mgrB* mutation (violet) analyzed in this study. The number of isolates was displayed on the left axis. The gray bars show the colistin MIC distribution from the EUCAST ECOFF (*n* = 1841) database (6) with the number of isolates displayed on the right axis.

All ST258 isolate genomes (previously sequenced using an Illumina MiSeq instrument [5]) were analyzed for the presence of polymyxin resistance determinants. The *mcr* resistance genes were not detected in any of the isolates, and none of two-component-systems, namely, *phoPQ* and *crrAB*, carried mutations. The *pmrB* mutation R256G was present in all ST258s from our collection, including susceptible isolates. In *mgrB*, we identified a single nucleotide substitution, namely, 59G>C, leading to amino acid change W20S that was present in 93% (39/42) of the ST258 isolates and carried by the strains displaying the highest MICs (MIC distributions are shown in Fig. 1). Strains carrying MgrB W20S variants showed statistically higher polymyxin resistance levels than the EUCAST epidemiological cutoff (ECOFF) MIC panel distribution (6) (Wilcoxon-Mann-Whitney one-sided test, exact *P* = 2.2e-16; using the R Stats Package, version 4.0.3 [7]). We hypothesized that this mutation resulted in a nonfunctional MgrB leading to an increase in polymyxin MICs.

To confirm its role in polymyxin resistance, we performed complementation experiments in the following three ST258 K. pneumoniae isolates carrying the *mgrB*W20S mutation: two KPC-2-producers (P27 and HSP12) from the collection and one additional KPC-negative (P52) ST258 strain that was isolated from the same hospital. For genetic complementation, an apramycin resistance gene was amplified from pIJ773 (8) using the primers Xce-apra-F (5′-CCACATGTATCCGTCGACCTGCAGTTCG-3′) and apra-Xce-R (5′-CCACATGTGTGTAGGCTGGAGCTGCTTCG-3′). The resulting product was digested using the FastDigest restriction enzyme XceI (Invitrogen, Thermo Fisher Scientific Inc.) and cloned into pBluescript SK+ (Stratagene Inc.) to build pskA (9). The wild-type (WT) and mutant *mgr*B were amplified with its own promoter from the strains MGH78578 (*mgrB*-WT) and P52 (*mgrB*-W20S), respectively, using the primers Eco-*mgrB*-ext-F (5′-GGAATTCCTTAAGAAGGCCGTGCTATCC-3′) and *mgrB*-BamHI-ext-R (5′-CGGGATCCCGAAGGCGTTCATTCTACCACC-3′) adapted from Cannatelli et al. (10). After restriction with BamHI and EcoRI, the PCR products were cloned in pskA and their sequences were verified by Sanger sequencing (Fasteris, Geneva, Switzerland). Polymyxin B and colistin MICs were determined in triplicate by broth microdilution following EUCAST guidelines, using Escherichia coli ATCC 25922 as a quality control. To prevent spontaneous plasmid loss, MICs of all strains carrying pskA plasmid had to be determined in the presence of 25 μg/mL apramycin. Strains carrying the empty pskA plasmid were used as a control, and presence of the plasmid did not significantly influence the MICs (Table 1). The expression of *mgrB*-WT on the pskA plasmid was able to restore susceptibility to both polymyxin B and colistin in the three different strains harboring the chromosomal W20S mutation with more than 64-fold MIC reduction (Table 1). On the other hand, when the complementation was performed with *mgrB*-W20S, the strains remained resistant to polymyxins confirming that this MgrB variant was not functional.

**TABLE 1:** Polymyxin B and colistin MIC of complemented P27, P52, and HSP12 strains

Strain	MIC (μg/mL) of:		Interpretation
	Polymyxin B	Colistin	
P27	16	16	R
P27 + pskA-empty^*a*^	16	16	R
P27 + pskA-*mgrB*-WT^*a*^	≤0.25	≤0.25	S
P27 + pskA-*mgrB*-W20S^*a*^	16	8	R
P52	16	16	R
P52 + pskA-empty^*a*^	8	16	R
P52 + pskA-*mgrB*-WT^*a*^	≤0.25	≤0.25	S
P52 + pskA-*mgrB*-W20S^*a*^	8	16	R
HSP12	32	32	R
HSP12 + pskA-empty^*a*^	16	32	R
HSP12 + pskA-*mgrB*-WT^*a*^	≤0.25	≤0.25	S
HSP12 + pskA-*mgrB*-W20S^*a*^	8	16	R

a MIC in the presence of apramycin 25 μg/mL to circumvent plasmid loss.

This description of a deleterious mutation in MgrB at position W20 is in agreement with a recent biochemical functional analysis of MgrB in Escherichia coli showing that W20 is a key residue for a MgrB/PhoQ interaction (11). Its role in polymyxin resistance was not characterized because in E. coli *mgrB* plays a minor role compared with the acquisition of *mcr* (12). Two K. pneumoniae isolates carrying a *mgrB* W20R mutation have been reported previously, but this mutation was not further investigated (3, 13). Based on our results, we can speculate that the W20R substitution is also a loss-of-function mutation influencing polymyxin MICs. The proposed EUCAST ECOFFs for colistin (2 μg/mL) did not fully discriminate isolates possessing a W20S *mgrB* mutation from the wild-type population; five (5/39) isolates were still classified as susceptible to colistin by the current breakpoint. It is possible that other factors interfered with MgrB/PhoPQ polymyxin resistance pathways in these isolates.

To investigate any fitness cost linked with a W20S mutation, growth curves were performed in triplicates after dilution (1/100) of the log-phase culture (at optical density [OD], 1) in 1 mL of fresh Mueller-Hinton broth (MHB), distributed in a 24-well plate under continuous shaking (180 rpm) at 37°C in the plate reader Infinite 200Pro (Tecan Trading AG, Switzerland). Doubling times were calculated for the three MgrB WT isolates and three randomly chosen W20S isolates. The W20S mutation did not impair the fitness of the tested strains since the generation times of the three strains with a W20S mutation (HSP12, 23.27 min; P27, 22.67 min; P52, 22.84 min) were similar to those of the strains with WT mgrB (HSP87, 23.87 min; P15, 23.71 min; P39, 22.02 min). Growth curves are shown in Fig. S1 in the supplemental material. The presence of a compensatory mutation explaining this absence of fitness cost could not be ruled out in our experiment. This observation is in line with previous publications showing that MgrB inactivation does not impose a fitness cost both in mutant isogenic strains and in the context of a clinical outbreak (14–16).

Here, we reported and confirmed by genetic experiments a novel amino acid substitution leading to polymyxin resistance in clinical high-risk K. pneumoniae isolates. This KPC-2-producing ST258 subclone carrying the W20S mutation was able to disseminate locally provoking 39 bloodstream infections over a 3-year period (2014 to 2016) (4). The heavy usage of polymyxin B in this hospital, in both empirical and directed therapies, due to a high rate of carbapenem-resistant isolates, might play a role in the efficient dissemination of this clone. In a previous study, we showed that the polymyxin B resistance rate has increased dramatically in this hospital, raising from 0% to 30.6% between the years 2009 and 2015 among 224 K. pneumoniae isolates recovered from blood cultures (17). The local dissemination of this polymyxin-resistant variant of the major international clone ST258 is thus worrisome, justifying that specific attention is needed to detect this new polymyxin resistance determinant.

### Data availability.

Sequences are available under BioProject accession numbers PRJNA510003, PRJNA628956, PRJNA629307, PRJNA628957, PRJNA629309, PRJNA628954, PRJNA628953, and PRJEB41225.
