# Supplementary material for: Characterization of Amino Acid Substitution W20S in MgrB Involved in Polymyxin Resistance in Klebsiella pneumoniae
Source: Microbiol Spectr. 2022 Feb 16;10(1):e01766-21. doi: 10.1128/spectrum.01766-21 (PMC8849082; doi:10.1128/spectrum.01766-21)
Supplement: SUPPLEMENTAL FILE 1 — Supplemental material. Download SPECTRUM01766-21_Supp_1_seq4.pdf, PDF file, 0.8 MB [file spectrum01766-21_supp_1_seq4.pdf]

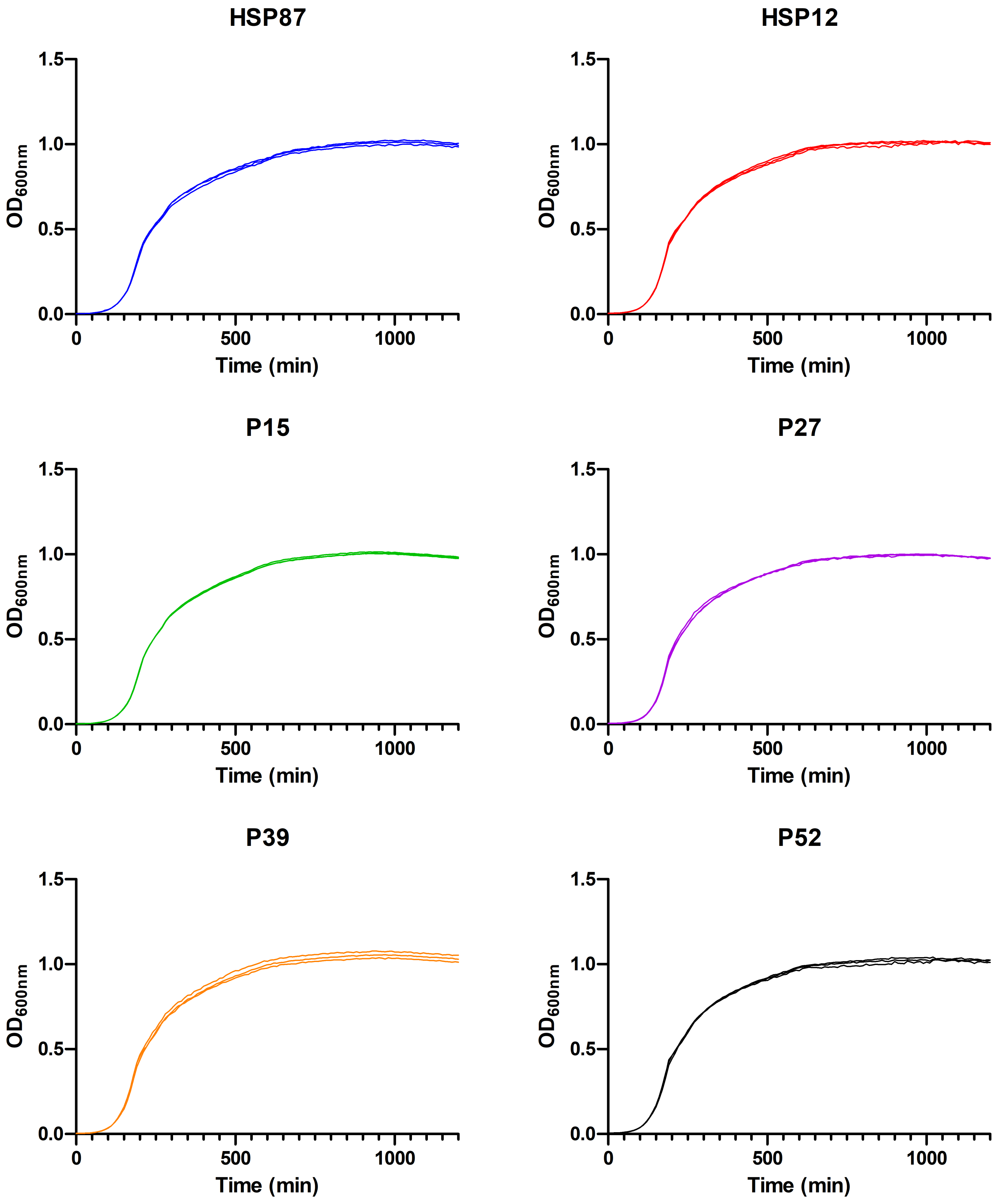

Supplementary Figure 1: Growth curves in triplicate of three isolates harboring the W20S mutation (HSP12, P27, P52) and three isolates with mgrB WT (HSP87, P15, P39). No difference of generation time was observed.

Supplementary Table 1 : Characteristics of the isolates and clinical data associated

Strain	Carbapenemase	16S-methylase	Polymyxin	Colistin	MgrB	Patient age	Patient sex	Infection source	Treatment	30-day outcome
HSP01	*bla*$_{KPC-2}$	*rmtB*	>64	>64	W20S	50	M	VAP	MER	ALIVE
HSP07	*bla*$_{KPC-2}$	*rmtB*	16	16	W20S	53	M	CLABSI	PMB+MER+RIF	DEAD
HSP08	*bla*$_{KPC-2}$	*rmtB*	2	2	W20S	39	M	CLABSI	[illegible]MB+MER+AMK+TIG+F[illegible]	DEAD
HSP16	*bla*$_{KPC-2}$	*rmtB*	32	64	W20S	MD[b]	MD	MD	MD	DEAD
HSP22	*bla*$_{KPC-2}$	*rmtB*	8	4	W20S	30	M	CLABSI	MER+GEN	DEAD
HSP31	*bla*$_{KPC-2}$	*rmtB*	8	8	W20S	36	M	CLABSI	PMB+MER+GEN	DEAD
HSP61	*bla*$_{KPC-2}$	*rmtB*	4	4	W20S	59	F	SSTI	PMB+MER+AMK	DEAD
HSP78	*bla*$_{KPC-2}$	*rmtB*	16	8	W20S	53	M	CLABSI	MER	DEAD
HSP86	*bla*$_{KPC-2}$	*rmtB*	2	2	W20S	32	M	ABDOMINAL	PMB+MER+AMK	ALIVE
HSP87	*bla*$_{KPC-2}$	*rmtB*	0.5	0.25	WT	43	F	UTI	PMB+MER+ERT	ALIVE
P15	*bla*$_{KPC-2}$	*rmtB*	0.5	1	WT	60	F	SSTI	PMB+MER+AMK	DEAD
P27	*bla*$_{KPC-2}$	*rmtB*	16	4	W20S	62	F	CNS	PMB+MER	DEAD
P37	*bla*$_{KPC-2}$	*rmtB*	4	8	W20S	57	M	VAP	PMB+MER+AMK	ALIVE
P38	*bla*$_{KPC-2}$	*rmtB*	1	2	W20S	60	M	CLABSI	PMB+GEN+MER+IMI	DEAD
P39	*bla*$_{KPC-2}$	*rmtB*	0.25	0.25	WT	65	M	MBI-LCBI	PMB+MER	DEAD
P43	*bla*$_{KPC-2}$	*rmtB*	8	8	W20S	61	F	CLABSI	PMB+MER	DEAD
P46	*bla*$_{KPC-2}$	*rmtB*	4	8	W20S	55	F	CLABSI	MER	ALIVE
P47	*bla*$_{KPC-2}$	*rmtB*	32	64	W20S	77	F	VAP	PMB+MER+AMK	ALIVE
P48	*bla*$_{KPC-2}$	*rmtB*	64	64	W20S	27	M	CLABSI	MER+AMK	ALIVE
P52[a]	Absent	*rmtB*	8	16	W20S	MD	MD	MD	MD	MD
P54	*bla*$_{KPC-2}$	Absent	32	64	W20S	35	M	VAP	PMB+MER+AMK	DEAD
P55	*bla*$_{KPC-2}$	*rmtB*	8	4	W20S	36	M	SSTI	PMB+MER+AMK	ALIVE
P57	*bla*$_{KPC-2}$	*rmtB*	4	8	W20S	72	M	CLABSI	CEF	ALIVE
P58	*bla*$_{KPC-2}$	*rmtB*	2	2	W20S	41	F	PNEUMONIA	PMB+MER+AMK	DEAD
P59	*bla*$_{KPC-2}$	*rmtB*	32	64	W20S	71	F	CLABSI	PMB+MER+AMK	ALIVE
P61	*bla*$_{KPC-2}$	*rmtB*	32	16	W20S	71	F	CLABSI	PMB+MER+TIG	ALIVE
P68	*bla*$_{KPC-2}$	*rmtB*	8	16	W20S	58	F	CLABSI	PMB+MER+AMK	ALIVE
P69	*bla*$_{KPC-2}$	*rmtB*	4	4	W20S	76	M	VAP	PMB+MER+AMK	DEAD
P71	*bla*$_{KPC-2}$	*rmtB*	8	4	W20S	55	F	CNS	MER	DEAD

P72	*bla* $_{KPC-2}$	*rmtB*	4	8	W20S	63	F	VAP	PMB+MER+GEN	DEAD
P73	*bla* $_{KPC-2}$	*rmtB*	16	32	W20S	67	M	CLABSI	PMB+MER	ALIVE
P74	*bla* $_{KPC-2}$	*rmtB*	4	4	W20S	85	M	SSTI	PMB+MER+AMK	DEAD
P75	*bla* $_{KPC-2}$	*rmtB*	4	4	W20S	78	F	CLABSI	MER+GEN	ALIVE
P76	*bla* $_{KPC-2}$	*rmtB*	4	8	W20S	73	M	CLABSI	PMB+MER+AMK	ALIVE
P78	*bla* $_{KPC-2}$	*rmtB*	8	8	W20S	54	M	CLABSI	PMB+MER+AMK	DEAD
P79	*bla* $_{KPC-2}$	*rmtB*	8	16	W20S	84	M	VAP	MER+ERT+TIG	DEAD
P80	*bla* $_{KPC-2}$	*rmtB*	2	4	W20S	70	M	VAP	CAZ-AVI	DEAD
P83	*bla* $_{KPC-2}$	*rmtB*	4	4	W20S	71	M	VAP	PMB+MER+AMK	DEAD
P86	*bla* $_{KPC-2}$	*rmtB*	8	16	W20S	42	F	VAP	PMB+MER+AMK	ALIVE
P87	*bla* $_{KPC-2}$	*rmtB*	8	16	W20S	74	M	ABDOMINAL	PMB+MER	DEAD
P88	*bla* $_{KPC-2}$	*rmtB*	2	2	W20S	57	M	SSTI	PMB+MER+AMK+TIG	DEAD
P89	*bla* $_{KPC-2}$	*rmtB*	4	4	W20S	83	F	ABDOMINAL	PMB+MER+ERT+TIG	DEAD
P90	*bla* $_{KPC-2}$	*rmtB*	8	8	W20S	64	F	CLABSI	MD	ALIVE

PMB, polymyxin B; MER, meropenem; ERT, ertapenem; IMI, imipenem; CEF, ceftriaxone; CAZ-AVI, ceftazidime-avibactam; AMK, amikacin; GEN, gentamicin; TIG, tigecycline; FOS, fosfomycin
VAP, ventilator-associated pneumonia; CLABSI, central-line associated bloodstream infection; SSTI, skin and soft tissue infection;
UTI, urinary tract infection; CNS, central nervous system; MBI-LCBI, mucosal barrier injury laboratory-confirmed bloodstream infection

[a] Clinical data were not available for the non-carbapenemase producing strain P52

[b] MD, missing data
